# Stem cell therapy for inherited retinal diseases: a systematic review and meta-analysis

**DOI:** 10.1186/s13287-023-03526-x

**Published:** 2023-10-05

**Authors:** Xiaodong Chen, Ningda Xu, Jiarui Li, Mingwei Zhao, Lvzhen Huang

**Affiliations:** 1 Department of Ophthalmology, Peking University People’s Hospital, Eye Diseases and Optometry Institute, Beijing, China; 2 Beijing Key Laboratory of Diagnosis and Therapy of Retinal and Choroid Diseases, Beijing, China; 3 College of Optometry, Peking University Health Science Center, Beijing, China

**Keywords:** Stem cell, Inherited retinal diseases (IRD), Retinitis pigmentosa (RP), Stargardt disease (STGD), Meta-analysis

## Abstract

**Purpose:**

Stem cell therapy is a promising therapeutic approach for inherited retinal diseases (IRDs). This study aims to quantitatively examine the effectiveness and safety of stem cell therapy for patients with IRDs, including retinitis pigmentosa and Stargardt disease (STGD).

**Methods:**

We searched PubMed, EMBASE, Web of Science, Cochrane Library databases, and the ClinicalTrials.gov website. The latest retrieval time was August 20, 2023. The primary outcomes were rates and mean difference (MD) of best-corrected visual acuity (BCVA) improvement. Subgroup analyses were conducted according to administration routes and stem cell types. This study was registered with PROSPERO (CRD42022349271).

**Results:**

Twenty-one prospective studies, involving 496 eyes (404 RP and 92 STGD) of 382 patients (306 RP and 76 STGD), were included in this study. For RP, the rate of BCVA improvement was 49% and 30% at 6 months and 12 months, respectively, and the BCVA was significantly improved in the operative eyes at 6 months post-treatment (MD = − 0.12 logMAR, 95% CI .17 to − 0.06 logMAR; *P* < 0.001), while there was no significant difference at 12 months post-treatment (MD = -0.06 logMAR; 95% CI − 0.13 to 0.01 logMAR; *P* = 0.10). For STGD, the rate of BCVA improvement was 60% and 55% at 6 months and 12 months, respectively, and the BCVA was significantly improved in the operative eyes at 6 months (MD = − 0.14 logMAR, 95% CI − 0.22 to − 0.07 logMAR; *P* = 0.0002) and 12 months (MD = − 0.17 logMAR, 95% CI − 0.29 to − 0.04 logMAR; *P* = 0.01). Subgroup analyses showed suprachoroidal space injection of stem cells may be more efficient for RP. Eleven treated-related ocular adverse events from three studies and no related systemic adverse events were reported.

**Conclusions:**

This study suggests stem cell therapy may be effective and safe for patients with RP or STGD. The long-term vision improvement may be limited for RP patients. Suprachoroidal space injection of stem cells may be a promising administration route for RP patients. Limited by the low grade of evidence, large sample size randomized clinical trials are required in the future.

## Introduction

Inherited retinal diseases (IRDs) are a group of complex and heterogeneous diseases that are mainly characterized by progressive photoreceptors (PRs) and/or loss of retinal pigment epithelium (RPE) cells, eventually leading to irreversible vision loss [1]. It is estimated that approximately 1 in 2000 to 4000 people are affected by IRDs [2, 3]. IRDs such as retinitis pigmentosa (RP) and Stargardt disease (STGD) have become the most common cause of blindness in the working-age population (16–64 years) in some Western countries [4, 5], which would impair the life quality of patients [6, 7], and cause severe social economic burden [8, 9]. In the past, only genetic testing and low-vision rehabilitation were used for the management of IRDs and these could not effectively slow or stop vision loss of patients with IRDs. However, recent emerging treatments including gene therapy, stem cell therapy, and retinal prosthesis have entered the stage of clinical trials and some therapies have shown inspiring therapeutic benefits in these vision-threatening disorders [10].

Among these treatment approaches for IRDs, stem cell therapy is considered a potential therapeutic method, which aims to replace lost cells in the retina with stem cells, mainly for those patients with IRDs who remained some useful retinal ganglion cells. Several types of stem cells, including retinal progenitor cells (RPCs), mesenchymal stem cells (MSCs), human embryonic stem cells-derived RPE (hESCs-RPE) cells, and induced pluripotent stem cells-derived RPE (iPSCs-RPE) cells, have been examined their efficacy or safety for IRDs patients in clinical trials [11].

Despite some results with small sample sizes from reviewed trials showing effectiveness and safety, no studies have been empowered to prove statistically significant efficacy for humans, and no stem cell therapy is approved for patients with IRDs [12]. The long-term efficacy and safety are controversial and required to be determined [13–17]. In addition, some parameters of stem cell therapy for IRDs, such as administration routes and types of transplanted stem cells, are needed to optimize [18]. To date, no systematic review or meta-analysis has quantitatively examined the effectiveness of vision improvement and adverse events of stem cell therapy for patients with IRDs. Therefore, this study aims to quantitatively assess these outcomes of stem cell therapy for patients with IRDs including RP and STGD and perform subgroup analyses stratified by administration routes and stem cell types in RP.

## Methods

The protocol of this study was registered at PROSPERO (CRD42022349271, [http://www.crd.york.ac.uk/PROSPERO/]). This meta-analysis was performed according to the guidelines of the Preferred Reporting Items for Systematic Reviews and Meta-Analyses (PRISMA 2020 statement) [19].

### Search strategy

We searched the PubMed, EMBASE, Web of Science, and Cochrane Library databases. We also screened the references from retrieved papers and the ClinicalTrials.gov website to identify additional related clinical studies and unpublished studies with available data. The following literature search terms were used (“Stem cell” OR “stem cells” OR “progenitor cell” OR “bone marrow”) AND (“Inherited retinal diseases” OR “inherited retinal degeneration” OR “hereditary retinal diseases” OR “inherited retinal dystrophy” OR “retinitis pigmentosa” OR “Stargardt disease” OR “Stargardt macular dystrophy”). Neither the article language nor the retrieval time was limited. The latest retrieval time was August 20, 2023.

### Inclusion and exclusion criteria

The inclusion criteria were as follows:

1. Patients who are diagnosed with IRDs, including RP and STGD.
2. Patients who have undergone stem cell therapy.
3. Any clinical trials.

The exclusion criteria were as follows:

1. Patients with other ocular disease except RP or STGD.
2. Preclinical studies, letters to the editor, editorials, case reports, conference abstracts, and reviews.
3. Studies without the assessment of primary outcome.

### Data extraction

Two researchers independently screened titles and abstracts according to the eligible criteria. All discrepancies were resolved through adjudication by a third researcher. Extracted information included author name, publication year, country, study design, number of participants, number of treated eyes, follow-up time, age, gender, diagnosis, stem cells, administration routes, and cell concentration. For studies that reported similar results, only the most complete publication was included. The improvement of best-corrected visual acuity (BCVA) and ocular and systemic adverse events related to stem cell therapy were examined. The primary outcomes were the rate and mean difference (MD) of improvement of BCVA measured in the logarithm of the Minimum Angle of Resolution (logMAR).

### Quality assessment

The Newcastle–Ottawa Quality Assessment Scale (NOS) was used to assess the risk of bias in each cohort study [20]. Two researchers independently evaluate the quality of studies. All discrepancies were resolved through adjudication by a third researcher.

### Statistical analysis

Meta-analyses were conducted using the Review Manager (version 5.3; Cochrane Collaboration) and Stata SE (version 15.1). Visual acuity values were recorded as Snellen or logMAR, and Snellen values were converted to logMAR for analyses. LogMAR values corresponding to count fingers (CF), hand movements (HM), and light perception (PL) were substituted with 2.0, 3.0, and 4.0 logMAR, respectively, in accordance with the previous study [21]. Besides, no light perception (NLP) was substituted with 5.0. Snellen values provided in studies were converted to logMAR equivalents [22]. For studies provided The Early Treatment Diabetic Retinopathy Study (ETDRS) letter scores, we converted them to logMAR equivalents using the following formula logMAR = 1.7–(0.02) * (ETDRS letter scores) [23]. The mean difference (MD) and 95% confidential interval (CI) were used to calculate. A fixed-effects model was used to assess the pool effect of changed logMAR, when no significant heterogeneity was detected (*I*^2^ ≤ 50% or *P*-value for heterogeneity ≥ 0.1). Otherwise, a random-effects model was used (*I*^2^ > 50% or p-value for heterogeneity < 0.1). Subgroup analyses were performed stratified by types of stem cells and administration routes. The publication bias was detected using funnel plots and Begg’s test. The statistical significance was set at *P* < 0.05.

## Results

### Literature search

**Fig. 1:**
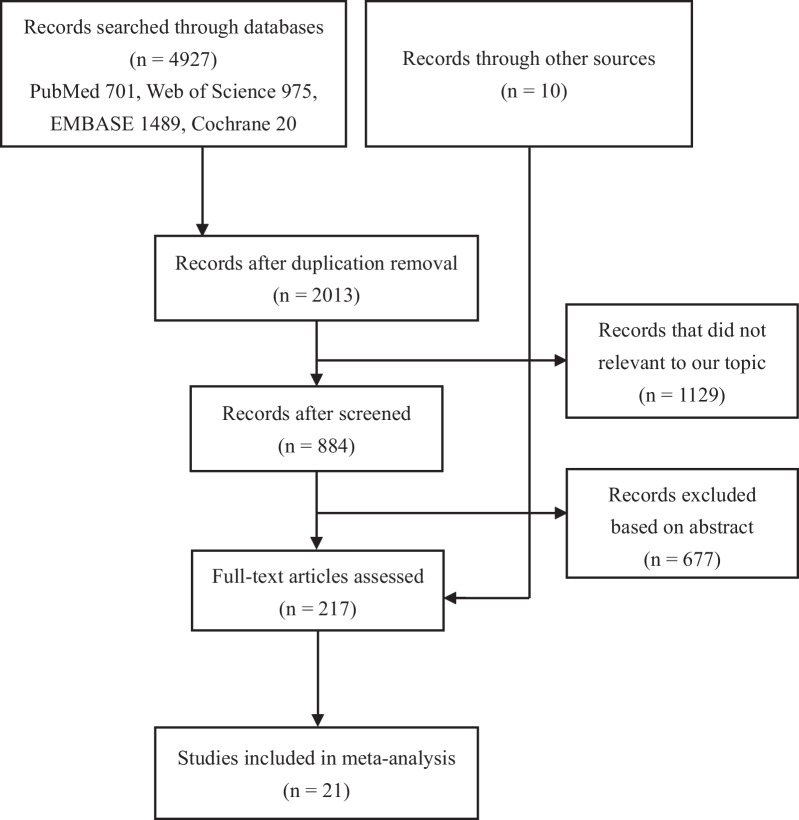
The selection process of included studies

The initial retrieval identified 2013 nonduplicated articles. Records that were not relevant to our topic were excluded (*n* = 1129). After screening titles and abstracts, studies were excluded according to inclusion criteria (*n* = 677). Full-text evaluation was performed in the remaining 217 studies. Ultimately, 21 unique studies were included in this meta-analysis (Fig. 1).

### Characteristics of included studies

Twenty-one prospective studies, involving 496 eyes (404 RP and 92 STGD) of 382 patients (306 RP and 76 STGD), were included in this study [14, 15, 24–42]. One study included RP and STGD patients [25]. Eight studies were for STGD [26, 28, 29, 36–38, 40, 42], and twelve studies were for RP [14, 15, 24, 27, 30–35, 39, 41], of which one study involved pediatric patients [41]. The mean follow-up duration was 14.4 ± 12.8 months (RP: 10.5 ± 5.0 months; STGD: 19.1 ± 18.1 months), ranging from 6 to 60 months (5 years). Detailed characteristics of the included studies are presented in Table 1.

**Table 1:** Study characteristics

References	Study design	Country	Patients	Female (%)	eyes	Age	Diagnosis	Stem cells	Administration routes	Follow-up	Quality scores
Siqueira et al., [24]	Nonrandomized PCT	Brazil	5	2 [40]	5	Mean (SD) [range] 31.4 (5.0) [23–35]	RP or cone-rod dystrophy	BM with CD34 + SCs	Intravitreal	10	6
Park et al., [25]	PCT	USA	6	2 (33.3)	6	Mean (SD) [range] 48.3 (25.7) [23–85]	Advanced RP or STGD or AMD or CRAO	BM with CD34 + SCs	Intravitreal	6	6
Schwartz et al., [26]	PCT	USA	18	5 (27.8)	18	Median (IQR) STGD: 50 (20–71) AMD: 77 (70–88)	STGD or AMD	hESC-RPE (MA09)	Subretinal	22	8
Liu et al., [37]	PCT	China	8	5 (62.5)	8	Mean (SD) [range] 35.5 (12.1) [19–53]	Advanced RP	RPCs	Subretinal	24	8
Weiss et al., [38]	Nonrandomized PCT	USA	17	7 (41.2)	33	Mean (SD) [range] 48.8 (13.9) [28–70]	RP	BMSCs	SCOT combination	6	7
Oner et al., [41]	PCT	Turkey	8	3 (37.5)	8	Mean (SD) [range] 44.8 (23.6) [19–75]	STGD or AMD	ADMSCs	Suprachoroidal	6	6
Mehat et al., [29]	PCT	UK	12	1 (8.3)	12	Mean (SD) [range] 45.3 (5.3) [34–53]	STGD	hESC-RPE	Subretinal	12	8
Oner et al., [30]	PCT	Turkey	14	5 (35.7)	14	Mean (SD) [range] 39.1 (8.9) [26–57]	Severe RP	ADMSCs	Subretinal	12	7
Özmert and Arslan, [31]	PCT	Turkey	32	14 (43.8)	34	Median [range] 38.7 [18–58]	RP	WJMSCs	Subtenon	6	6
Kahraman and Oner, [32]	PCT	Turkey	82	32 (39.0)	124	Median (IQR) 38.5 (34.0–46.0)	RP	UCMSCs	Suprachoroidal	6	5
Limoli et al., [33]	PCT	Italy	25	11 (44.0)	34	Mean (SD) [range] 45.9 (18.4) (19–86)	RP	ADMSCs	Suprachoroidal	6	7
Zhao et al., [34]	PCT	China	32	20 (62.5)	64	Mean (SD) [range] 36 (2.5) [16–61]	Advanced RP	UCMSCs	Intravenous	12	6
Tuekprakhon et al., [15]	Nonrandomized PCT	Thailand	14	5 (35.7)	14	Mean (SD) [range] 46.2 (9.3) [32–61]	Advanced RP	BMSCs	Intravitreal	12	7
Wiącek et al., [35]	Nonrandomized PCT	Poland	30	18 (60.0)	30	Mean (SD) [range] 41.7 (12.8) [19–64]	RP	BM-derived Lineage-negative cells	Intravitreal	12	6
Sung et al., [36]	Nonrandomized PCT	Korea	3	0 (0.0)	3	Mean (SD) [range] 41.7 (2.89) [40–45]	STGD	hESC-RPE (MA09)	Subretinal	36	7
Li et al., [37]	PCT	China	7	5 (71.4)	7	Mean (SD) [range] 23.3 (3.6) [19–27]	STGD	hESC-RPE	Subretinal	60	8
Weiss et al., [38]	PCT	USA	17	5 (29.4)	34	Mean (SD) [range] 48.2 (16.1) [26–72]	STGD	BMSCs	SCOT combination	12	8
Khairullah et al., [39]	PCT	Malaysia	2	1 (50.0)	4	Mean (SD) [range] 61.0 (5.7) [57–65]	Advanced RP	WJMSCs	Subtenon	12	4
Fernandes et al., [40]	PCT	Brazil	12	9 (75.0)	12	Mean (SD) [range] 41.5 (7.1) [30–53]	STGD	hESC-RPE	Subretinal	12	8
Oner et al., [41]	PCT	Turkey	46	18 (39.0)	46	Median (IQR) 13.4 (9.0–17.0)	RP	UCMSCs	Suprachoroidal	12	6
Cotrim et al, [42]	Nonrandomized PCT	Brazil	10	6 (60.0)	10	Mean (SD) [range] 33.0 (8.6) [23–48]	STGD	BMMF with CD34 + SCs	Intravitreal	6	7

PCT Prospective clinical trial, IQR Interquartile range, *CRAO* Central Retinal Artery Occlusion, *RP* Retinitis pigmentosa, *STGD* Stargardt disease, *AMD* Age-related macular degeneration, *RPCs* Retinal progenitor cells, *BMSCs* Bone marrow-derived mesenchymal stem cells, *ADMSC* Adipose tissue-derived mesenchymal stem cells, *UCMSCs* Umbilical cord mesenchymal stem cells, *hESC-RPE* Human embryonic stem cell-derived retinal pigment epithelium, *SCOT combination* The Stem Cell Ophthalmology Treatment Study, *BMMF* Bone Marrow Mononuclear Fraction, *SCOT* used various administration routes to treat

### The rate of best-corrected vison acuity improvement after stem cell therapy

For RP, 49% and 30% operative eyes achieved better BCVA at 6 months and 12 months post-treatment, respectively. For STGD, 60% and 55% operative eyes achieved better BCVA at 6 months and 12 months post-treatment, respectively (Table 2).

**Table 2:** The rate of the best-corrected visual acuity improvement after stem cell therapy at 6 months and 12 months

References	Improved operative eyes at 6 months	Total operative eyes at 6 months	Improved operative eyes at 12 months	Total operative eyes at 12 months
*RP*				
Siqueira et al., [24]	3	3	NA	NA
Park et al., [25]	1	1	NA	NA
Liu et al., [26]	5	8	3	8
Weiss et al., [27]	15	33	NA	NA
Oner et al., [28]	3	11	NA	NA
Özmert and Arslan, [31]	32	34	NA	NA
Limoli et al., [33]	25	34	NA	NA
Kahraman and Oner, [41]	57	124	NA	NA
Wiącek et al., [35]	16	30	17	30
Zhao et al., [34]	12	64	11	64
Khairullah et al., [39]	0	2	0	2
Total	169	344	31	104
Rate		49%		30%
*STGD*				
Park et al., 2015	2	2	NA	NA
Cotrim et al., [42]	8	10	NA	NA
Fernandes et al., [40]	10	12	10	12
Li et al., [37]	2	7	1	6
Mehat et al., [29]	4	12	5	12
Oner et al., [28]	4	4	NA	NA
Schwartz et al., [26]	3	8	3	7
Sung et al., [36]	2	3	3	3
Total	35	58	22	40
Rate		60%		55%

*NA* Not available; *RP* Retinitis pigmentosa; *STGD* Stargardt disease

### Improvement in best-corrected visual acuity after stem cell therapy

**Fig. 2:**
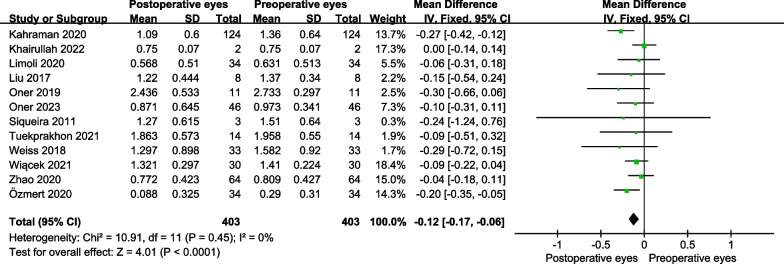
The forest plot showed the best-corrected visual acuity improvement for patients with RP at 6 months

**Fig. 3:**
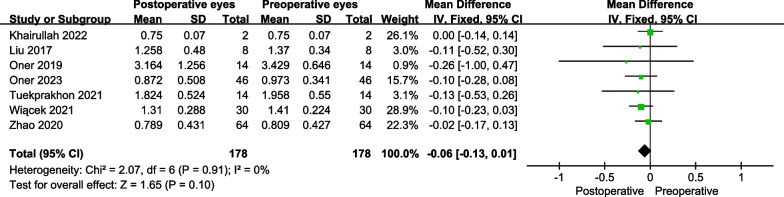
The forest plot showed the best-corrected visual acuity improvement for patients with RP at 12 months

For RP, the BCVA was significantly improved in the operative eyes at 6 months post-treatment (MD = − 0.12 logMAR, 95% CI − 0.17 to − 0.06 logMAR; *P* < 0.001) (Fig. 2), while there was no significant difference at 12 months post-treatment (MD = 0.06 logMAR; 95% CI − 0.13 to 0.01 logMAR; *P* = 0.10) (Fig. 3).

**Fig. 4:**
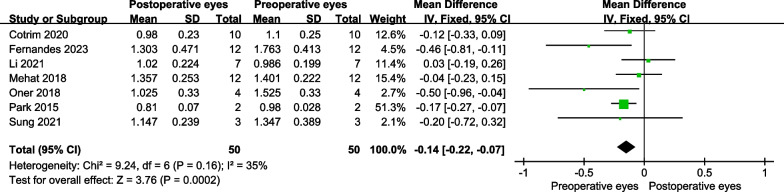
The forest plot showed the best-corrected visual acuity improvement for patients with STGD at 6 months

**Fig. 5:**
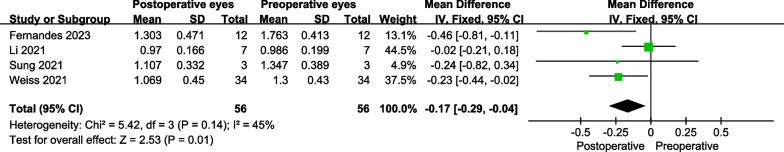
The forest plot showed the best-corrected visual acuity improvement for patients with STGD at 12 months

For STGD, the BCVA was significantly improved in the operative eyes at 6 months post-treatment (MD = − 0.14 logMAR, 95% CI − 0.22 to − 0.07 logMAR; *P* = 0.0002) (Fig. 4) and 12 months (MD = − 0.17 logMAR, 95% CI − 0.29 to − 0.04 logMAR; *P* = 0.01) (Fig. 5).

### Subgroup analyses

**Fig. 6:**
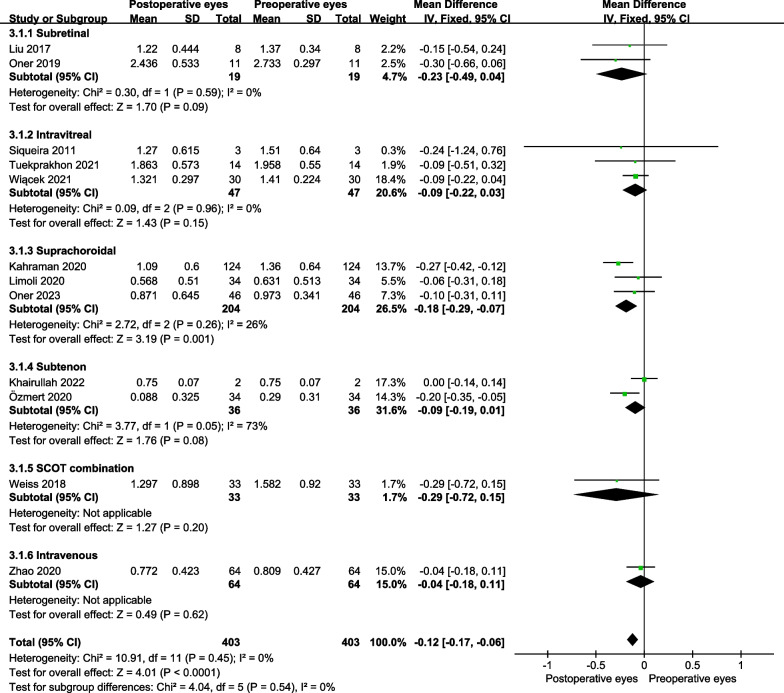
Subgroup analyses showed different administration routes on the best-corrected visual acuity improvement for RP at 6 months

**Fig. 7:**
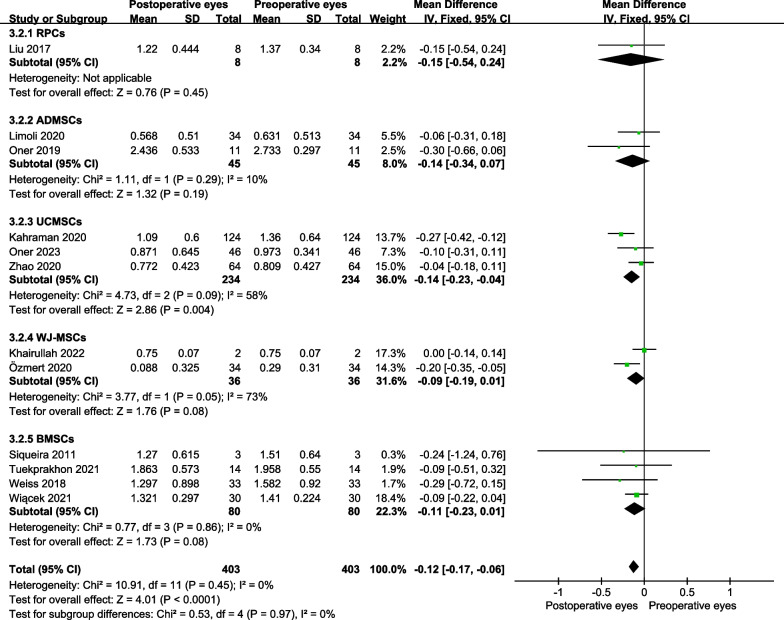
Subgroup analyses showed different stem cell types on the best-corrected visual acuity improvement for RP at 6 months

For improvement in BCVA of RP patients at 6 months post-treatment, we performed subgroup analyses according to the administration routes and types of stem cells. For administration routes, suprachoroidal space injection showed the best BCVA improvement at 6 months post-treatment (MD = − 0.18 logMAR, 95% CI − 0.29 to − 0.07 logMAR; *P* = 0.001) (Fig. 6). For types of stem cells, umbilical cord MSCs (UCMSCs) injection showed the best BCVA improvement at 6 months post-treatment (MD = − 0.14 logMAR, 95% CI − 0.23 to − 0.04 logMAR; *P* = 0.004) (Fig. 7).

### Publication bias

**Fig. 8:**
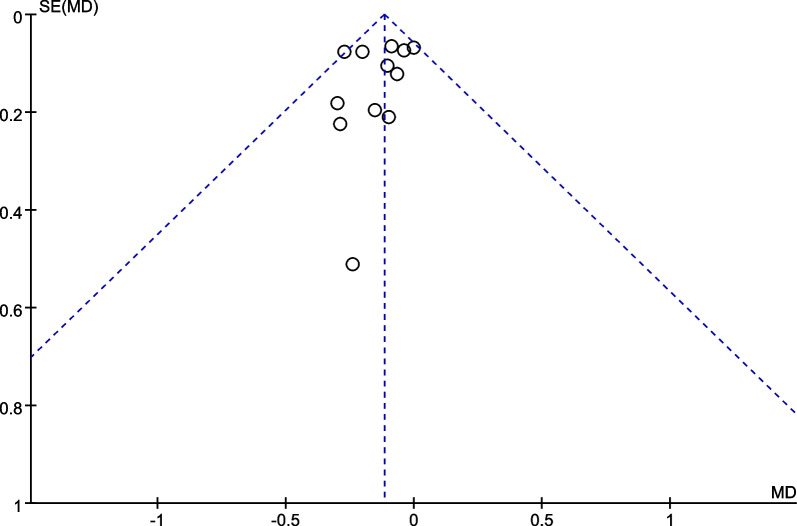
The funnel plot for the best-corrected visual acuity improvement for patients with RP at 6 months

**Fig. 9:**
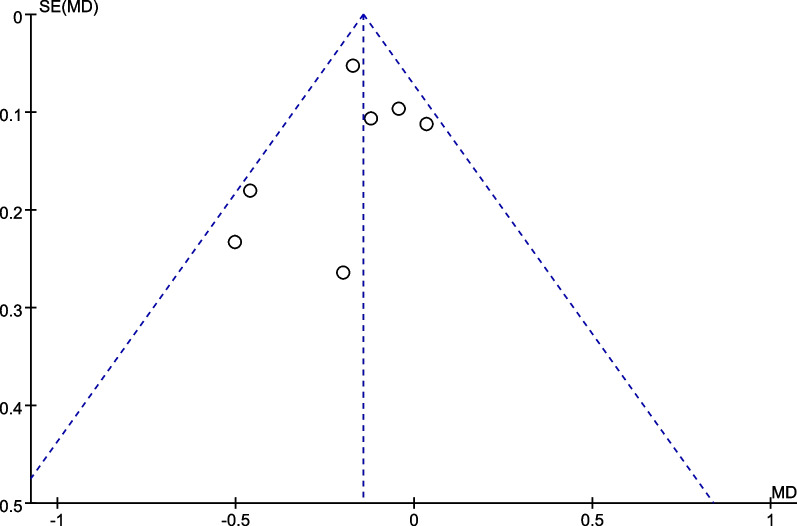
The funnel plot for the best-corrected visual acuity improvement for patients with STGD at 6 months

The funnel plots demonstrated the improvement in BCVA at 6 months after stem cell therapy for patients with RP (Fig. 8, P for Begg’s test: 0.170) and STGD (Fig. 9, P for Begg’s test: 0.652). No significant publication bias was detected.

### Systemic and ocular adverse events

Eleven treated-related ocular adverse events from three studies and no related systemic adverse events were reported for RP patients [15, 30, 35]. One study reported three tractional retinal detachments (RD) [35]. One study with long-term follow-up reported a case that experienced diffuse vitreous hemorrhage and osseous metaplasia in the ciliary body in the third year of follow-up and a case that developed minimal intraocular lens subluxation in the fourth year of follow-up [15]. Another study reported a case that developed choroidal neovascular membrane (CNM) and five cases had epiretinal membrane (ERM) with peripheral tractional RD [30].

## Discussion

Our study was in line with previous systemic reviews which confirmed stem cell therapy was an effective and relatively safe treatment for patients with RP or STGD [16, 43, 44]. This present study, including 21 studies and 496 eyes, was the first to quantitatively assess the improvement of BCVA in patients with RP or STGD who had undergone stem cell therapy. The change of logMAR of STGD patients was significantly improved at 6 and 12 months. However, although the BCVA of RP patients was significantly improved at 6 months, this improvement was no longer significant at 12 months. A study that used RPC cells to treat RP patients showed that vision improvement did not appear at 24 months after stem cell therapy [14]. In addition, another study used hESC-RPE cells to treat STGD patients showed worse BCVA at 60 months after stem cell therapy [37]. The incidence of adverse events after stem cell therapy was low, and most of them were mild ocular adverse events, but the safety of stem cell therapy for patients with RP or STGD requires attention. One study reported the first five cases developed peripheral tractional RD and one case happened CNM [30]. They considered these complications may be attributed to inadvertent preretinal injection of stem cells or reflux of transplanted stem cells from the subretinal space [45]. After modifying the surgical operation, the remaining patients did not have adverse events. Our results showed suprachoroidal space injection showed optimally improved logMAR at 6 months with no serious ocular or systemic adverse events reported, indicating this may be a better administration route of stem cell therapy for RP patients. The standardized surgical procedures were important to the safety of stem cell therapy. These findings suggest that stem cell transplantation is efficient and relatively safe for patients with RP or STGD, but long-term efficacy is uncertain for RP. Weiss et al. indicated that the efficacy of stem cell therapy would be affected by the severity of RP [27]. Meanwhile, the patients with longer disease duration gained less vision improvement, compared to those with shorter duration of RP [35]. In the meta-analysis showing the efficacy of stem cell therapy for RP at 12 months, five in seven studies were advanced RP. Marginally significant improvement was observed in the remaining two studies at 12 months (data not shown) [35, 41].

Stem cells have a strong ability to proliferate and differentiate into many kinds of cells, including RPE cells, PRs, and RGCs. The transplanted stem cells function mainly by secreting neurotrophic factors, replacing the degenerative cells in the host, upregulating anti-apoptotic genes, and forming new functional synapses [46]. In 2016, researchers proposed a new potential mechanism that host and grafted cells could happen material transfer to rescue the host degenerative retina [47], and this mechanism was further verified by subsequent studies [48, 49]. Despite the inspiring results in clinical trials, the exact mechanisms underlying stem cell therapy for IRDs are necessary to explore.

Currently, three common methods are applied to deliver stem cells into the eye: intravitreal injection, subretinal injection, and suprachoroidal injection. Intravitreal injection is a relatively simple and safe procedure, and this method is widely used for treating retinal diseases [50]. However, an intact blood-retinal barrier limited the transport of transplanted stem cells and stem cell-secreted neurotrophic factors [51]. Another serious problem is that the drug can diffuse to nontarget regions such as lens and subretinal space and then trigger fibrous tissue proliferation and lead to RD and ERM [17, 52]. Although some clinical studies have reported the general safety of stem cell therapy for RP patients, this method should be taken into rigorous consideration before being used [15, 35]. Subretinal injection aims to deliver stem cells to the potential space between RPE and PR, which can directly target the retina. Although this method involves a pars plana vitrectomy which may lead RD and vitrectomy-associated complications, the successful use of hESC-RPE in subretinal space has shown its relative safety when carefully using the right techniques [53]. The suprachoroidal space (SCS) is a potential space, between the choroid and sclera. SCS injection is a novel administration route to the posterior segment, which accurately targets the choroid, RPE, and neuroretina, with high bioavailability [54]. Limoli et al. first described the suprachoroidal implantation method of stem cells (they called it the Limoli Retinal Restoration Technique, LRRT) [55–57]. This method allows stem cell-produced growth factors to enter the choroidal blood flow. In this study, the exact mechanism underlying the better efficacy of SCS injection is not clear. One possible explanation is that no ocular adverse events, such as RD and ERM which can impair vision, were reported for the safe SCS injection, compared to intravitreal and subretinal injection. In addition, the accumulation and distribution of drug in the SCS can achieve sustained release [58], which may allow stem cell-derived growth factors to be constantly secreted to the choroid and retina. For those patients who need to inject cell suspension multiply, less invasive SCS inject may be a suitable administration route. For the emerging transplantation of stem cells sheet with the technique of tissue engineering for IRDs, subretinal injection is still the first choice [59].

In this present study, most RP patients were injected with MSCs. MSCs represent the most frequently studied type of adult stem cells, which are derived from stromal progenitor cells of mesodermal origin [60]. MSCs are found in various parts of the human body, and bone MSCs (BMSCs), adipose tissue-derived MSCs (ADMSCs), and UCMSCs are the three main MSCs used to research IRDs, and they have similar function properties [61, 62]. Several important properties of MSCs include immunomodulation, anti-inflammation, and secretion of neurotrophic factors [60]. Compared to other stem cells, easier isolation from tissues makes MSCs a promising candidate for IRDs. Besides, MSC-derived extracellular vesicles are considered beneficial to retinitis pigmentosa [63]. Our results showed that UCMSCs may be a potential MSCs type for patients with RP. Currently, clinical trials are focused on the transplantation of hESCs-RPE or iPSCs-RPE to treat retinal degeneration [18]. Both ESCs and iPSCs can be successfully differentiated into PRs, RPE cells, and other retinal cells and are seemed a promising way to treat IRDs [64]. In 2012, Schwartz et al. first reported the preliminary results using hESC-RPE to treat two retinal diseases including age-related macular degeneration (AMD) and STGD [65]. Subsequently, a plethora of clinical trials showed inspiring results of hESC for treating RP [11] and STGD [44]. However, ethical concerns limit the use of hESCs; thus, iPSCs are considered a potential alternative to avoid the above problems. In 2006, Takahashi and Yamanaka first discovered iPSCs, which can be derived from embryonic or adult fibroblasts in mouse by introducing four transcription factors [66]. Then, they described this type of stem cells can be obtained from human skin fibroblasts and peripheral blood in 2007 [67]. Like hESCs, iPSCs were soon reported to be able to differentiate into retinal cells in vitro in 2009 [68]. In 2011, milestone study investigated a self-organized 3D optic cup and stratified RPE from mouse iPSCs, creating the research field of retinal organoids [69]. In 2012, Li et al. reported a method to obtain and transplant iPSC-RPE cells into RP mouse model, which was considered a pioneering study on the use of iPSC in the field of retinal diseases [70]. In 2014, RIKEN reported the first clinical trial using autologous iPSCs-RPE to treat a patient with AMD; the vision of this patient was not improved or worsened [71]. Then the first clinical trial using iPSC-retina which was prepared from retinal organoids to treat advanced RP was started by Kobe City Eye Hospital in 2020 [72]. Despite some challenges, scientific researchers spare no effort to pave the way for the practical application of stem cell therapy for patients suffering from retinal degeneration [59, 73].

Some limitations exist in this study. First, we did not evaluate the data from fundus autofluorescence, electroretinogram, and optical coherence tomography, because the sample size was small or these data could not be extracted and synthesized for meta-analysis. Second, subgroup analyses were only performed in RP patients at 6 months after stem cell therapy. Besides, the number of studies in each subgroup was small in subgroup analyses. Third, the definition of serious ocular events differs and lacks standardized criteria.

## Conclusions

This study suggests stem cell therapy may be effective and safe for patients with RP or STGD. The long-term vision improvement may be limited for RP patients. Suprachoroidal space injection of stem cells may be a promising administration route for RP patients. Limited by the grade of evidence, large sample sizes and well-designed multi-center randomized controlled trials with long follow-up periods are required in the future.

## Data Availability

Data sharing is not applicable to this article as no datasets were generated or analyzed during the current study.
